# Detailed assessment of the hemodynamic response to psychosocial stress using real-time MRI

**DOI:** 10.1186/1532-429X-13-S1-P68

**Published:** 2011-02-02

**Authors:** Alexander Jones, Jennifer A Steeden, Jens C Pruessner, John E Deanfield, Andrew M Taylor, Vivek Muthurangu

**Affiliations:** 1 UCL Institute of Child Health, London, UK; 2 Douglas Institute, Deparment of Psychiatry, McGill University, Montreal, QC, Canada

## Introduction

Mental stress is a potent stimulator of the cardiovascular system and has been linked to a number of cardiovascular diseases. Characterization of the cardiovascular response to mental stress is crucial for understanding the underlying mechanisms. Previous studies have relied upon unreliable stress paradigms and limited cardiovascular parameters, with mixed results. An experimental method that causes stress reliably and allows accurate and detailed measurement of cardiovascular physiology during it, holds the promise to significantly further understanding of the emerging links between stress and cardiovascular disease.

## Purpose

To demonstrate that combining the Montreal Imaging Stress Task (MIST) with real-time cardiac MR allows detailed assessment of the cardiovascular mental stress response.

## Methods

22 healthy volunteers (1:1 M:F, 26-64 years) underwent MR imaging during rest and the MIST. Real-time spiral phase contrast MR, accelerated with sensitivity encoding (SENSE) was used to assess stroke volume (SV) and radial k-t SENSE was used to assess ventricular volumes. Simultaneous heart rate (HR) and blood pressure (BP) measures allowed calculation of cardiac output (CO), systemic vascular resistance (SVR) and arterial compliance (TAC). Endocrine responses were assessed using salivary cortisol.

## Results

In response to stress, BP increased due to increased CO and reduced TAC but not increased SVR, which fell (see Figure). HR, not SV, determined CO increases. Men had greater BP during stress (130 vs 118 mmHg; *P*=.04) due to greater CO increases and relatively higher SVR. Older participants had greater BP responses (r=.47, *P*=.03) due to greater falls in TAC (r=-.45, *P*=.04). Greater cortisol response was correlated with greater falls in TAC (r=-.59, *P*=.006) but resting cortisol and TAC were not related (r=-.32, *P*=.17).

**Figure 1:**
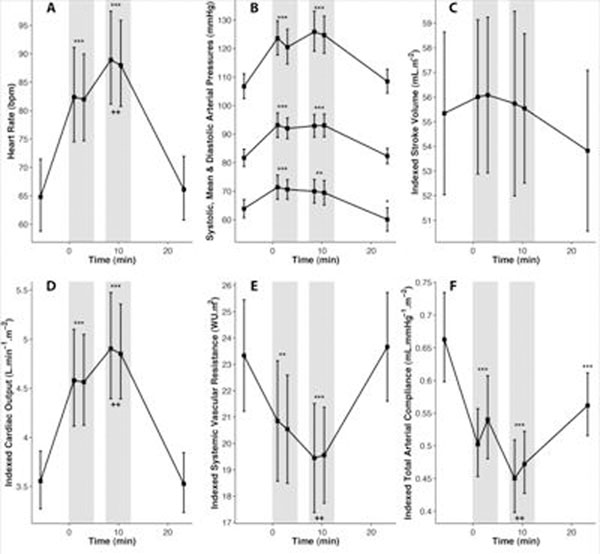
Mean ± 95% CI values (geometric values for A, D, F & systolic arterial pressure in B) at rest and in response to two 5-minute periods of stress (vertical grey bars) for (A) heart rate; (B) systolic, mean and diastolic arterial pressure; (C) stroke volume indexed to body surface area (BSA); (D) cardiac output indexed to BSA; (E) systemic vascsular resistance indexed to BSA and (F) total arterial compliance indexed to BSA. *P <0.0125, **P<0.0025, ***P<0.00025 for pair-wise 2-sided *t*-test comparisons of mean values during each stress period, or the recovery period, with those at reast. **P<0.0025 for pair-wise 2-sided *t*-test comparisons of values during first and second stress periods. *P*-value thresholds were Bonferroni corrected to account for four comparisons per variable.

## Conclusions

This new approach allows detailed, accurate and reliable assessment of stress physiology. Preliminary findings suggest stress exposes relationships, not seen at rest, of cardiovascular function with age, sex and endocrine function.

